# Promoting Healthy Behaviors among Egyptian Mothers: A Quasi-Experimental Study of a Health Communication Package Delivered by Community Organizations

**DOI:** 10.1371/journal.pone.0151783

**Published:** 2016-03-18

**Authors:** Angela Brasington, Ali Abdelmegeid, Vikas Dwivedi, Adrienne Kols, Young-Mi Kim, Neena Khadka, Barbara Rawlins, Anita Gibson

**Affiliations:** 1 Maternal and Child Survival Program, Save the Children, Washington, DC, United States of America; 2 Maternal and Child Survival Program, Jhpiego, Washington, DC, United States of America; 3 Maternal and Child Survival Program, John Snow, Inc., Boston, Massachusetts, United States of America; 4 Jhpiego, Baltimore, Maryland, United States of America; Nathan Kline Institute and New York University School of Medicine, UNITED STATES

## Abstract

Decisions made at the household level, for example, to seek antenatal care or breastfeed, can have a direct impact on the health of mothers and newborns. The SMART Community-based Initiatives program in Egypt worked with community development associations to encourage better household decision-making by training community health workers to disseminate information and encourage healthy practices during home visits, group sessions, and community activities with pregnant women, mothers of young children, and their families. A quasi-experimental design was used to evaluate the program, with household surveys conducted before and after the intervention in intervention and comparison areas. Survey questions asked about women’s knowledge and behaviors related to maternal and newborn care and child nutrition and, at the endline, exposure to SMART activities. Exposure to program activities was high in intervention areas of Upper Egypt: 91% of respondents reported receiving home visits and 84% attended group sessions. In Lower Egypt, these figures were 58% and 48%, respectively. Knowledge of danger signs related to pregnancy, delivery, and newborn illness increased significantly more in intervention than comparison areas in both regions (with one exception in Lower Egypt), after controlling for child’s age and woman’s education; this pattern also occurred for two of five behaviors (antenatal care visits and consumption of iron-folate tablets). Findings suggest that there may have been a significant dose-response relationship between exposure to SMART activities and certain knowledge and behavioral indicators, especially in Upper Egypt. The findings demonstrate the ability of civil society organizations with minimal health programming experience to increase knowledge and promote healthy behaviors among pregnant women and new mothers. The SMART approach offers a promising strategy to fill gaps in health education and counseling and strengthen community support for behavior change.

## Introduction

Pregnant women and young children in Egypt face persistent health challenges. Neonatal mortality has been declining more slowly than under-five mortality [1]and now accounts for 52% of all under-five deaths in Egypt [2]. Malnutrition also remains a concern, manifesting itself in low birth weight and stunting [2,3]. Choices made at the household level—for example, when and how often to go for antenatal care, whether to consume iron and folic acid (IFA) tablets during pregnancy, how long to breastfeed exclusively, and when to seek care for a sick child—influence health outcomes [4]. Although health services are widely available and utilized in Egypt, there is little emphasis on effective counseling and other communication to improve maternal and newborn health behaviors [5].

Families in Egypt frequently make unhealthy decisions because they lack accurate information, do not feel confident in their ability to act, or think that others will disapprove of their actions. Only 21% of married women who responded to the 2008 Egypt Demographic and Health Survey were knowledgeable about danger signs during pregnancy and childbirth [3]. The inability to recognize danger signs and assess the seriousness of illness can lead to life-threatening delays by mothers in seeking health care for themselves and their newborns [6,7]. Norms and traditions also play an important role in decision-making. A 2013 cross-sectional study in Mansoura, Egypt found that 58% of newborns were given liquids other than breast milk before starting to breastfeed; the most frequent reasons were tradition and advice from mothers and mothers-in-law [8]. A case-control study in Cairo highlights the health impacts of sub-optimal behaviors by caretakers. A multivariate analysis found that the risk of malnutrition among children age 6–23 months was independently associated with five factors; not being exclusively breastfed increased the risk five times, and late initiation of breastfeeding, reluctance to seek medical advice during illness, and not attending health or nutrition education sessions each doubled the risk [9].

Comprehensive reviews of strategies to improve maternal and newborn health have concluded that community-based interventions encouraging healthy behaviors and appropriate utilization of health services can be an effective way to reduce morbidity and mortality [4,10]. Although women are often the focus of interventions to improve maternal and newborn health, they make decisions within the larger context of family and community. Women’s choices are influenced by social networks that convey behavioral norms, health information, social support, and other resources that impact women’s social capital [11]. Therefore, health promotion interventions may have a greater impact if they encompass the broader community rather than focusing on individuals [12].

In Egypt, the SMART Community-based Initiatives program adopted an approach aimed at both individual women and the influencers of their decisions, with the goal of improving neonatal health and child nutrition outcomes. The program worked with community development associations (CDAs) to conduct community health outreach and communication activities in both Upper and Lower Egypt. This paper assesses the impact of SMART activities on knowledge and behaviors related to pregnancy and newborn care among mothers of young children. The analysis answers the following questions:

1. How effective was the intervention package in reaching mothers of young children?
2. Did mothers’ knowledge and behaviors on maternal and newborn care and child nutrition improve significantly in intervention areas? Was improvement greater in intervention than comparison areas?
3. In intervention areas, was there a dose-response relationship between exposure to SMART activities and mothers’ knowledge and behaviors?

## Materials and Methods

### Study Design and Sample

The intervention could only be implemented in areas where there were well-functioning CDAs and no security problems, making it impossible to conduct a randomized trial. Therefore, the program evaluation employed a quasi-experimental design. Household surveys were conducted before the intervention, in September-October 2012, and after the end of the program, in January-February 2014, in both intervention and comparison areas.

**Fig 1:**
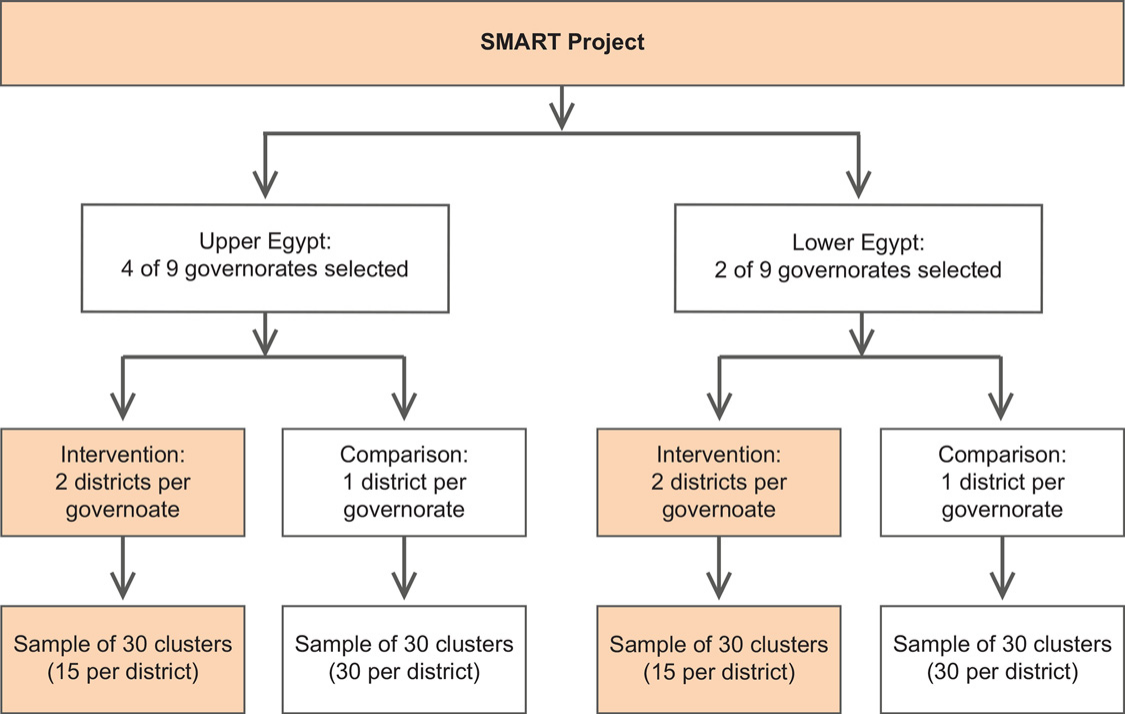
Sampling scheme for selection of districts.

Fig 1 shows the sampling scheme for the study. Six of Egypt’s 27 governorates were purposefully selected to implement the SMART intervention because of a relatively high poverty level and poor health indicators. Special attention was given to chronic malnutrition, but data on poverty, migration return, contraceptive prevalence, pregnancy spacing, teenage pregnancy, antenatal care, deliveries by skilled providers, and maternal mortality were also considered in the selection process. Since there are strong regional differences in Egypt, the evaluation was designed to assess the impact of the program separately in Upper and Lower Egypt. Of the program’s six priority governorates, four were located in Upper Egypt (Asyut, Beni Suef, Qena and Sohag) and two in Lower Egypt (Qalyubia and Sharqia). In each governorate, two districts with active, experienced CDAs were purposefully selected to implement the intervention, and one comparison district, matched to the intervention districts on population size, wealth, and CDA activity level, was selected; this yielded a total of 12 intervention and six comparison districts. Fewer comparison than intervention districts were included in order to reduce study costs and channel more resources into the intervention. Although the number of districts was different for comparison and intervention groups, the sample size was equal for each of the four strata: Upper Egypt intervention, Upper Egypt comparison, Lower Egypt intervention, and Lower Egypt comparison.

Multi-stage cluster sampling methods were used to select survey respondents. Within each of the four strata, 30 clusters (each equivalent to a village) were randomly selected based on population proportionate to size (PPS) sampling methodology. In each cluster, the survey team followed “next-door” sampling methodology [13] to identify 53 households with a mother who had a child under age two. If there was more than one eligible mother in a household, one was randomly selected to be interviewed. If a mother had more than one child under age two, the interview focused on the youngest child.

This analysis uses a subsample of the survey: mothers who had children less than one year of age at the time of the survey. Because these women were pregnant and/or had infants during the 12- to 14-month duration of program activities, they were most likely to have substantial exposure to the intervention. Table 1 shows the number of respondents included in the analysis. A post-hoc power analysis was performed based on the primary outcome indicator (proportion of women with knowledge of at least 3 newborn danger signs), which had a baseline prevalence of 15% with an intra-cluster coefficient (icc) of 0.10. Assuming a type I error of 0.05, power of 80%, the smallest strata sample size of 757 respondents (30 clusters with an average of 25 respondents per cluster) would have been adequate to detect differences of 11% for an increasing outcome and 8% for a decreasing outcome.

**Table 1:** Number of Respondents included in the Analysis, by Survey Round, Study Group, and Region.

Region	Baseline		Endline	
	Intervention group	Comparison group	Intervention group	Comparison group
Upper Egypt	798	840	877	881
Lower Egypt	804	757	802	885
Total	1,602	1,597	1,679	1,766

Note: Analysis was limited to women with children age 0–11 months.

### Description of the Intervention

The SMART program worked through local organizations, community health workers (CHWs), and local service providers to roll out a set of effective interventions covering life stages from conception to age two. In each intervention district, SMART partnered with an established umbrella CDA with roots in the district and a proven track record in managing donor funds; most had limited experience in the health sector. Each umbrella CDA identified and supported five to 10 local CDAs (one per village) to implement SMART activities and helped recruit and train CHWs. Twelve umbrella CDAs, 100 local CDAs, and 1,200 CHWs implemented the SMART intervention package between November 2012 and November/December 2013 in 100 villages with a total population of more than 2 million, including approximately 57,000 pregnant women and 112,000 children under age two [14].

Female CHWs recruited from within the community formed the backbone of the program. The hiring process was competitive, and women had to demonstrate a desire to serve the community. All CHWs had at least a secondary education; most were married; and many had experience with development projects, but not necessarily with health initiatives. Local CDAs recruited 12 CHWs to cover the catchment area of a single primary health unit, which consisted of one village and, in some cases, nearby hamlets; the CHWs divided this area up by neighborhood based on social mapping. CHWs worked 30 to 35 hours a week for a monthly stipend of 300 Egyptian pounds (US $42), less than half what government outreach health workers earned. Their retention rate was 98% [14]. During monitoring visits and focus groups, CHWs said they were motivated by their desire to give back to the community and the opportunity to gain new knowledge and skills; they also appreciated the community recognition and improved social status that came with their role as a CHW [15].

The program provided a training of trainers in health communication to interested local private-sector service providers. Some subsequently conducted the training for CHWs, including 10 days of classroom instruction and five days of field-based training. The training covered best practices in health care and nutrition for new mothers and young children; topics included birth preparedness, breastfeeding, food selection, and growth monitoring. Participants engaged in role plays and hands on activities, with special emphasis placed on developing counseling and education skills. They received a reference manual covering health topics and an operational manual covering the timing and content of home visits and group sessions.

Two senior CHWs, who had greater experience and additional training, supervised every 10 CHWs; CHWs met at least weekly with their peers to discuss problems, seek advice, and refine plans. Village Health Committees oversaw the CHWs; members included religious leaders, business people, teachers, clinicians, and other respected community members. Program staff also conducted random monitoring visits to ensure the quality of their work.

**Fig 2:**
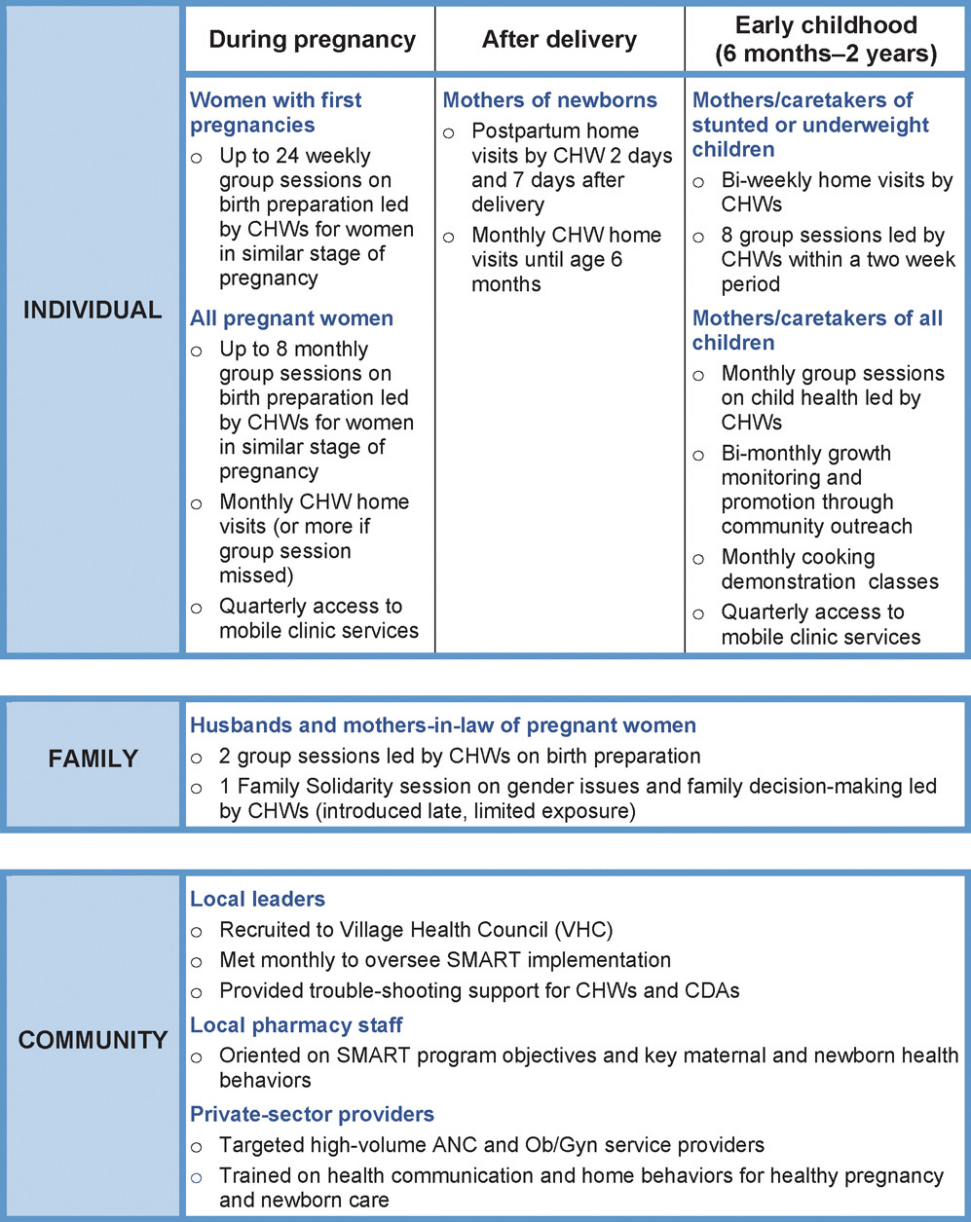
SMART intervention package: Activities at the individual, family, and community levels.

SMART intervention activities were conducted at three different levels (Fig 2). The most intensive activities occurred at the individual level, with CHWs promoting behavior change among pregnant women and mothers; intended outcomes included timely and appropriate care seeking, planning for facility delivery, dietary changes for women and infants, and adoption of family planning. At the family level, SMART activities were directed to husbands and mothers-in-law in order to establish an enabling environment for women to practice recommended behaviors. Less intensive activities at the community level targeted local leaders, pharmacy staff, and health care providers in order to reinforce CHWs’ messages and contribute to an enabling environment for women to act.

Each CHW was responsible for identifying all women who were pregnant or had children under age two in her catchment area, which typically included 200 to 350 households and a total population of around 2,000. CHWs were asked to make monthly home visits to each of these women to provide counseling. They also invited women to attend monthly group counseling sessions with women at a similar stage of pregnancy or motherhood and other community events, such as bi-monthly weighing of infants and cooking classes. Home visits and group sessions were scheduled more frequently for women with first pregnancies, pregnant women at higher risk because of their medical or personal history, and underweight children. CHWs also helped conduct family solidarity sessions with mothers-in-law and husbands, during which they discussed gender issues and family decision-making. During home visits and group sessions, CHWs provided emotional support and information on antenatal, postnatal, and infant care; offered nutrition counseling and growth monitoring; and discussed how culture and equity issues can affect health and well-being. Village Health Committee members, local pharmacists, and doctors reinforced messages disseminated by CHWs.

The SMART intervention was designed to complement, not supplant, the existing service delivery system so CHWs were limited to counseling and referrals. The Ministry of Health collaborated on deploying mobile clinics in all participating villages, with CDAs covering their operating costs. At the time of the study, shortages of commodities and physicians meant that village-level primary health units were often unable to offer a full range of services. The mobile clinics offered maternal and child health, family planning, and internal medicine services and could refer women to higher level health facilities outside the village.

### Data Collection and Analysis

Interviewers and supervisors received five days of training on informed consent and confidentiality, sampling techniques, recruitment, interviewing, and recording answers. Data were collected using an instrument based largely on the 2008 Egypt Demographic and Health Survey (EDHS) questionnaire. Knowledge questions covered side effects of IFA tablets, danger signs during pregnancy, danger signs during and after delivery, and danger signs for newborn illness. Behavior questions covered antenatal care (ANC) visits, consumption of IFA tablets, place of delivery, presence of a skilled birth attendant, initiation of breastfeeding, and what the child was fed during the last 24 hours. Other questions gathered data on the socio-demographic characteristics of the women and the households in which they lived. At endline, mothers were asked how many and what kinds of home visits they had received from SMART CHWs, how often they had attended group sessions, and whether their husband had participated in a men’s session during the six months preceding the survey.

Data were cleaned prior to analysis by checking completed questionnaires, and a wealth index was constructed based on self-reported asset ownership [16]. The survey samples were first placed within the national distribution of household wealth. Then we identified asset and household variables common to the SMART survey and the 2008 EDHS 2008; 19 binary variables met this criterion. Principal component analysis (PCA) scores were obtained from 2008 EDHS, and a sensitivity analysis of the reduced set of variables was conducted. After calculating asset scores with the variables common to both surveys, we created an asset score for households in the SMART survey, standardizing each variable against the DHS distribution and multiplying these variables by the EDHS eigenvalue. The final step was to assign each household in the SMART survey to a wealth quintile according to the cut-off values retained from the 2008 DHS wealth quintiles.

A Chi-square test was used to assess regional differences in exposure to SMART activities. In this analysis, the independent variables were the time of data collection (baseline or endline), study group (intervention or comparison), and the interaction of these two terms. The interaction term tests for the difference in slope for the change in outcome scores from baseline to endline between the intervention and comparison groups (difference in differences). Bivariate and multivariable logistic regression analyses were performed for independent variables of interest with adjustment for clustering due to survey design. Clustering was accounted for by the use of robust variance estimators based on a first-order Taylor series linear approximation [17]. Confidence intervals at the 95% level are presented where applicable. The initial bivariate analysis calculated the gain or decline in outcome variables from baseline to endline separately for the intervention and comparison groups.

To answer the research question on dose-response effects, we created a seven-point scale to measure the intensity of women’s self-reported exposure to program activities. It included a maximum of three points for home visits, three points for women’s attendance at group sessions, and one point for husband’s attendance at a family solidarity session. Husband’s attendance was underweighted because these sessions did not begin until the last few months of the intervention. The more frequently women received home visits or attended group sessions, the more points were assigned; home visits more than once a month received three points as did weekly attendance at group sessions. Women were divided into three groups based on the intensity of their exposure to the intervention: low (0–1 point), medium (2–4 points), and high (5–7 points). Cronbach’s alpha was calculated to assess the internal reliability of the items included in the construct for program intensity; it measured 0.71, which exceeds the 0.70 threshold for acceptability [18]. Dose-response relationships between program intensity and changes in knowledge and behaviors for targeted interventions were analyzed using logistic regression, and the adjusted means are presented. All analyses were performed using Stata 12.0 SE.

### Ethical Considerations

This study was approved by the Johns Hopkins Bloomberg School of Public Health Institutional Review Board and the Research Ethics Committee (REC) at the Egyptian Society for Healthcare Development. They approved the use of oral rather than written consent because many study participants were illiterate or had limited literacy. Prior to each interview, oral informed consent was obtained from all women age 18 or older. For women under age 18, informed consent was obtained from the husband, who in Egypt is considered to be her legal guardian. Interviewers documented oral consent by signing and dating a form for each woman that indicated that she or her husband were informed about the study and had given consent to participate.

## Results

### Characteristics of Respondents

The sample included 3,199 women at baseline and 3,445 women at endline. In both Upper and Lower Egypt, a majority of respondents were aged 20–29, had at least 11 years of schooling, and had one or two children (Table 2). There were significant differences in education between intervention and comparison groups at baseline, but they were not consistent across regions. The intervention group was significantly wealthier than the comparison group at endline in Lower Egypt; that difference is discussed in the limitations section. About half of respondents in Upper Egypt were in the bottom two wealth quintiles, compared with less than one-third of respondents in Lower Egypt; this is consistent with regional economic differences in Egypt.

**Table 2:** Socio-demographic Characteristics of Respondents, According to Region and Study Group.

Characteristic	Upper Egypt						Lower Egypt					
	Baseline			Endline			Baseline			Endline		
	Inter-vention	Com-parison	p-value	Inter-vention	Com-parison	p-value	Inter-vention	Com-parison	p-value	Inter-vention	Com-parison	p-value
	(n = 798)	(n = 840)		(n = 877)	(n = 881)		(n = 804)	(n = 757)		(n = 802)	(n = 885)	
**Age**												
<20	7.4	7.5	0.582	5.5	5.5	0.805	8.6	6.5	0.194	8.2	8.8	0.342
20–29	61.3	60.8		61.4	62.5		67.1	68.4		69.8	67.2	
30–39	25.1	23.7		29.1	27.0		18.9	21.4		20.5	22.2	
40–49	1.3	2.4		2.0	3.0		1.8	0.9		1.0	1.8	
Missing	5.0	5.6		2.2	2.0		3.6	2.9		0.5	0.0	
**Years of schooling**												
None	17.6	14.5	0.034	24.5	31.2	0.123	10.0	15.4	<0.001	9.7	13.3	0.125
1–5	3.8	6.9		4.0	2.8		3.7	4.9		6.1	4.2	
6–10	14.8	16.9		17.9	13.1		9.4	8.6		15.0	8.8	
11+	61.9	56.7		53.6	49.8		66.6	69.2		69.2	73.6	
Missing	1.9	5.0		0.0	3.1		10.3	1.9		0.0	0.1	
**Number of living children**												
1–2	NA	NA		54.2	53.1	0.393	NA	NA		61.4	61.9	0.329
3–4				33.6	32.0					34.9	32.8	
5+				12.1	14.8					3.7	5.3	
**Wealth quintile**												
Poorest	NA	NA		28.6	30.7	0.499	NA	NA		4.9	13.1	<0.001
Poorer				21.2	23.2					12.5	19.7	
Middle				20.1	19.4					16.3	22.5	
Richer				16.4	19.0					25.9	20.0	
Richest				13.7	7.8					40.4	25.6	

### Exposure to SMART Activities in Intervention Areas

The program reached more women, more intensively, in Upper than Lower Egypt. Forty-five percent of women in Upper Egypt received home visits more than once a month, and the same proportion attended group sessions weekly, compared with 17.1% and 12.4%, respectively, in Lower Egypt (p<0.001). In Upper Egypt, only 8.8% of women never received a home visit, and 16% never participated in a group session, compared with 41.8% and 52.3% in Lower Egypt (Table 3).

**Table 3:** Percent Distribution of Women in the Intervention Group at Endline by Participation in Program Activities.

Activity	Upper Egypt	Lower Egypt	p-value
	(n = 877)	(n = 802)	
***Home Visits by SMART CHWs***			
**Frequency of home visits in the 6 months preceding the survey**			
More than once a month	45.3	12.4	<0.001
Once a month	31.0	34.4	
Less than once a month	14.9	11.5	
None	8.8	41.8	
**Number of ANC counseling and referral visits received during last pregnancy**			
Four or more	77.5	68.5	<0.001
Two or three visit	16.7	17.3	
One visit	2.7	8.9	
None	3.1	5.4	
**Any postnatal care counseling and referral visit**			
Yes	81.2	48.1	<0.001
No	18.8	51.9	
***Group Sessions***			
**Frequency of women’s participation in group sessions in the 6 months preceding the survey**			
Once a week	44.9	17.1	<0.001
Once a month	28.9	23.7	
Less than once a month	10.1	7.0	
None	16.1	52.3	
**Husband participated in at least one session (reported by wives)**			
Yes	16.1	3.6	<0.001
No	83.9	96.4	

A large majority of women in intervention areas in both Upper and Lower Egypt (77.5% and 68.5%, respectively) reported at least four home visits for ANC counseling from SMART CHWs. But women in Upper Egypt were 1.7 times more likely to receive a postnatal care visit from a SMART CHW than women in Lower Egypt (81.2% versus 48.1%). Husbands were four times more likely to have attended a group session in Upper than Lower Egypt (16.1% versus 3.6%).

### Changes in Care-Seeking Knowledge and Behavior

Knowledge of danger signs during pregnancy, during and after delivery, and for newborn illness was extremely low at baseline in both Upper and Lower Egypt, never exceeding 20% (Table 3). In the intervention areas, gains in knowledge from baseline to endline were two to three times larger in Upper Egypt than Lower Egypt (Table 4). In contrast, knowledge levels changed little in comparison areas except for newborn illness. Differences between intervention and comparison areas were significant for all knowledge variables in Upper Egypt and all except one in Lower Egypt.

**Table 4:** Bivariate and multivariate analyses of changes in knowledge related to care seeking: Percentage of women who know at least three danger signs, by data collection round, study group, and region.

Knowledge of at least three danger signs:	UPPER EGYPT						LOWER EGYPT					
	Bivariate analysis				Multivariate analysis		Bivariate analysis				Multivariate analysis	
	Baseline	Endline	Change from baseline to endline within group		Adjusted p-value for change within group	p-value for inter-action	Baseline	Endline	Change from baseline to endline within group		Adjusted p-value for change within group	p-value for inter-action
	(n = 1,632)	(n = 1,740)	% points	p-value			(n = 1,574)	(n = 1,620)	% points	p-value		
**During pregnancy**												
Intervention group	11.6	56.2	44.6	<0.001	0.984	<0.001	12.3	25.7	13.4	0.010	0.235	0.037
Comparison group	11.9	14.0	2.1	0.529	<0.001		15.7	16.1	0.4	0.777	0.082	
**During delivery**												
Intervention group	4.9	46.1	41.2	<0.001	0.617	<0.001	6.6	19.2	12.6	<0.001	0.226	<0.001
Comparison group	5.7	11.6	5.8	0.152	<0.001		8.8	9.6	0.8	0.410	<0.001	
**After delivery**												
Intervention group	12.5	60.6	48.1	<0.001	0.529	<0.001	15.4	31.2	15.8	<0.001	0.337	0.010
Comparison group	14.3	15.0	0.3	0.949	<0.001		18.8	19.5	0.7	0.699	0.010	
**For newborn illness**												
Intervention group	9.5	67.0	57.5	<0.001	0.181	<0.001	11.0	39.7	28.7	<0.001	0.105	0.062
Comparison group	13.5	25.8	12.3	0.014	<0.001		16.4	29.8	13.4	<0.001	0.244	

Note: Danger signs during pregnancy include: vaginal bleeding, convulsions, severe abdominal pain, severe headache/blurring of vision, no fetal movements in more than 24 hours, fever, water leakage or vaginal discharge with foul smell. edema of hands and legs. Danger signs during delivery include: convulsions, high fever, heavy bleeding, fast/difficult breathing, retained placenta, headache/blurred vision, prolonged labor / severe delivery pains without progress for more than 12 hours, cord prolapse, water leakage for more than 12 hours without delivery of the baby. Danger signs after delivery include: excessive vaginal bleeding, fast/difficult breathing, high fever, severe pain and edema of leg calf, severe headache/blurred vision, convulsions/loss of consciousness, foul-smelling discharge from the vagina, severe pain and swollen breasts, verbalization/behavior that indicates woman may hurt herself or the baby. Danger signs for newborn illness include: convulsions, fever, poor suckling or feeding, fast/difficult breathing, baby feels cold (bluish skin), yellow palms/skin/eyes, swollen abdomen, baby does not urinate or defecate, unconscious, and pus or redness of the umbilical stump, eyes or skin

Only two of the five behavioral indicators showed significantly greater gains in intervention than comparison areas: making at least four ANC visits and consuming at least 90 IFA tablets (Table 5). The former was significant only in Upper Egypt. Intervention and comparison groups in both regions made similar gains on skilled birth assistance, exclusive breastfeeding, and dietary diversity.

**Table 5:** Bivariate and multivariate analyses of changes in behavior: Percentage of women with desired behavior, by data collection round, study group, and region.

Behavior	UPPER EGYPT						LOWER EGYPT					
	Bivariate analysis				Multivariate analysis		Bivariate analysis				Multivariate analysis	
	Baseline	Endline	Change from baseline to endline within group		Adjusted p-value for change within group	p-value for inter-action	Baseline	Endline	Change from baseline to endline within group		Adjusted p-value for change within group	p-value for inter-action
			% points	p-value					% points	p-value		
***Among women with children age 0–12 months*:**	***(n = 1*,*634)***	***(n = 1*,*740)***					***(n = 1*,*576)***	***(n = 1*,*620)***				
**Made at least 4 ANC visits**												
Intervention group	73.6	85.4	11.8	<0.001	0.175	<0.001	75.4	86.7	11.3	<0.001	0.016	0.263
Comparison group	78.3	71.7	-6.6	0.052	<0.001		82.7	89.3	6.6	<0.001	0.028	
**Consumed at least 90 IFA tablets** ^a^												
Intervention group	20.0	33.0	13.1	0.002	0.248	<0.001	21.4	36.1	14.7	<0.001	0.428	0.037
Comparison group	23.8	13.5	-9.8	<0.001	<0.001		23.5	29.4	5.4	0.358	0.003	
**Received skilled birth assistance**												
Intervention group	89.0	95.1	6.1	0.001	0.487	0.606	89.2	98.1	8.9	<0.001	0.032	0.854
Comparison group	90.8	96.4	5.6	0.135	0.079		93.0	98.8	5.8	<0.001	0.218	
***Among women with children age 0–5 months*:**	***(n = 706)***	***(n = 886)***					***(n = 688)***	***(n = 858)***				
**Breastfed exclusively**												
Intervention group	27.6	55.2	27.6	<0.001	0.795	0.711	37.1	57.8	20.7	<0.001	0.251	0.341
Comparison group	29.0	52.1	23.1	<0.001	0.782		28.9	57.1	28.2	<0.001	0.982	
***Among women with children age 6–11 months***	***(n = 928)***	***(n = 854)***					***(n = 888)***	***(n = 762)***				
**Fed 3+ dietary groups**												
Intervention group	5.4	18.8	13.4	<0.001	0.446	0.371	10.9	16.0	5.1	0.181	0.389	0.860
Comparison group	4.3	21.1	16.8	<0.001	0.618		8.5	13.8	5.3	0.040	0.602	

^a^ Information on IFA consumption is missing for 30 women at baseline and 12 women at endline in Upper Egypt and 33 women at baseline and 1 woman at endline in Lower Egypt.

### Dose-Response Relationship between Exposure and Outcomes

The findings suggest that knowledge of danger signs and certain behaviors may have increased with the intensity of respondents’ exposure to SMART activities, especially in Upper Egypt (Table 6). For example, knowledge of danger signs after delivery increased from 36.4% in the low-exposure group to 66.7% in the high-exposure group in Upper Egypt and from 21.3% to 49.6% in Lower Egypt. A similar pattern is observed for many other outcomes. However, the results are inconclusive; even where the p-values are significant, the confidence intervals for different exposure groups overlap. During the planning of the study, we did not plan for this kind of analysis and hence we are unable to infer if the lack of a statistical significance is due to sample size or lack of a detectable difference.

**Table 6:** Relationship between impact and exposure: Percentage of Women in the Intervention Group with Desired Knowledge or Behavior at Endline, by the Intensity of their Exposure to SMART Activities.

Indicator	Upper Egypt				Lower Egypt			
	Percent (CI)				Percent (CI)			
	Low	Medium	High	p-value	Low	Medium	High	p-value
**Knowledge of at least:**	**(n = 99)**	**(n = 256)**	**(n = 252)**		**(n = 320)**	**(n = 365)**	**(n = 117)**	
3 danger signs during pregnancy	33.3 (25.6–41.1)	56.6 (37.1–76.1)	64.3 (57.4–71.2)	0.017	24.7 (7.5–41.9)	23.6 (9.8–37.3)	34.2 (13.5–54.9)	0.291
3 danger signs during delivery	21.2 (9.5–32.9)	45.1 (30.9–59.2)	57.9 (45.3–70.6)	0.009	16.9 (13.2–20.6)	18.9 (12.9–24.8)	26.5 (5.1–47.8)	0.331
3 danger signs after delivery	36.4 (23.2–49.5)	62.4 (45.5–79.2)	66.7 (56.7–76.6)	0.016	21.3 (13.3–29.2)	34.0 (23.7–44.2)	49.6 (31.9–67.2)	0.001
3 danger signs of newborn illness	49.5 (35.1–63.9)	66.0 (49.8–82.1)	76.2 (65.6–86.8)	0.082	30.9 (10.7–51.5)	41.4 (22.7–60.0)	59.0 (31.4–86.6)	0.025
**Behavior**	**(n = 99)**	**(n = 256)**	**(n = 252)**		**(n = 320)**	**(n = 365)**	**(n = 117)**	
Made at least 4 ANC visits	75.8 (68.7–82.8)	86.5 (80.9–92.0)	86.9 (79.9–93.9)	0.012	83.1 (80.8–85.4)	87.7 (84.4–90.9)	93.2 (88.2–98.1)	0.001
Consumed at least 90 IFA tablets	15.2 (-0.00–30.7)	32.7 (23.3–42.1)	40.5 (28.2–52.7)	0.030	29.7 (23.4–35.8)	38.8 (33.2–44.3)	44.4 (27.4–61.5)	0.033
Received skilled birth assistance	95.0 (89.7–100)	95.4 (93.1–97.2)	94.4 (92.5–96.3)	0.699	97.5 (96.8–98.2)	98.1 (95.8–98.2)	100.0	0.134
***Among women with children age 0–5 months*:**	***(n = 50)***	***(n = 255)***	***(n = 128)***		***(n = 175)***	***(n = 193)***	***(n = 54)***	
Exclusively breastfed child in previous 24 hours	36.0 (12.5–59.5)	54.1 (39.2–69.1)	64.8 (46.1–83.5)	0.001	64.0 (54.1–73.9)	52.3 (38.7–66.1)	57.4 (45.9–68.9)	0.122
***Among women with children age 6–11 months*:**	***(n = 49)***	***(n = 271)***	***(n = 124)***		***(n = 145)***	***(n = 172)***	***(n = 62)***	
Fed child 3+ dietary groups in previous 24 hours	18.4 (3.7–33.0)	19.2 (13.0–25.3)	17.7 (9.8–25.6)	0.765	15.9 (9.4–22.2)	19.7 (10.4–29.2)	6.4 (0.1–12.1)	0.007

## Discussion

### Regional Differences in Program Reach

SMART activities reached most women who were pregnant or had young children. However, coverage was greater and exposure more intensive in Upper Egypt, which has long been a focus for development efforts because of high concentrations of poverty; the region is home to about half of the nation’s total population, but 87% of Egyptians who are extremely poor [19]. Probably because of their long experience with development projects and strong ties to the community, CDAs in Upper Egypt proved to be more efficient in selecting, training, and supporting CHWs and took extra steps in support of the program, such as forming a joint network to formalize knowledge- and experience-sharing [15]. Lack of employment opportunities for women in Upper Egypt also meant there was a large pool of motivated, educated candidates for CHWs, and Village Health Committees in these socially cohesive rural communities were eager to offer supervision and support for CHWs. Finally, women in Upper Egypt, whose mobility was limited, were receptive to and appreciative of CHW visits and opportunities to meet with peers in group sessions; the latter was only possible because of husbands’ and grandmothers’ trust in CHWs and their messages [15].

The contrasting setting in Lower Egypt, which is peri-urban and has a wealthier, more educated population [3,19], likely contributed to lower exposure to the intervention there. CDAs had less capacity and weaker ties to the community, and women had other job opportunities. CHWs generally covered larger catchment areas and were less likely to find women at home when they visited. Anecdotally, women in Lower Egypt were also less receptive to visits by CHWs, comparing their credentials unfavorably to private sector providers. In both regions, home visits reached more women than group sessions, presumably because they did not involve any effort on women’s part.

SMART activities in Upper Egypt reached more women, more intensively than most other community-based intervention packages reported in the literature, although differences in the interventions tested make detailed comparisons difficult [20–22]. Comparable programs in Africa and Asia have generally reached about one-half to two-thirds of women targeted, with low coverage often cited as a challenge [23–26]. Other programs typically planned two to seven home visits across the antenatal and neonatal period and group meetings every one to three months or even less frequently [22,23,25,27–31]. In contrast, in Upper Egypt the SMART program reached more than 90% of pregnant women and new mothers, 45% received home visits more than once a month; and 45% attended group sessions weekly. In Lower Egypt, the reach and intensity of SMART activities were closer to levels achieved by other programs.

### Program Intensity and Impact

The SMART program had a significant impact on maternal knowledge and, to a lesser extent, behavior, which is consistent with other studies. A series of systematic reviews and meta-analyses of community-based intervention packages promoting maternal and newborn health in low-resource settings have found that home visits and group activities have a positive impact on maternal knowledge of danger signs, care-seeking and service utilization, and desirable health practices such as early breastfeeding [22,32–36]. Data on health outcomes are not available for the SMART program, but randomized controlled trials and quasi-experimental studies have linked similar interventions with reductions in maternal and neonatal morbidity and mortality [10,25,30,33,34,36,37].

Our analysis suggests that there may be a dose-response relationship between SMART activities and women’s knowledge and behaviors, but the sample size was not adequate to draw a firm conclusion. This may be an important reason why the program showed greater impact in Upper Egypt where exposure to the intervention was more intensive. Evidence from other studies is mixed regarding the relationship between the intensity and impact of community-based intervention packages. For example, the number of group meetings attended was positively associated with care seeking and skilled delivery care in Bangladesh [27], but a systematic review looking across multiple studies found no consistent association between the number of home visits and impact on neonatal mortality [25]. Some reviews have concluded that interventions combining home visits and community mobilization activities have greater impact than interventions that rely on just one of these activities [10,36] and that high levels of community participation are important [35]. In Nigeria, however, a low-intensity approach utilizing only group discussions had just as much effect on most indicators of newborn and sick child care as a high-intensity approach that added home visits [32].

Simply counting the number of contacts may not be sufficient to measure program intensity. The nature and levels of community engagement are also important [35]. SMART activities reached out to husbands and mothers-in-law as well as women, brought neighbors together at community events, and engaged Village Health Committees and health care providers. The different interventions and contact points maximized social exposure to desirable behaviors. As described by Mead and colleagues [38], social exposure encompasses all of the environmental cues that shape a person’s ideas about what behaviors are prevalent and acceptable in their community or, in other words, normative. By creating new and consistent cues at the village level, our data suggest—and focus groups with local people confirm [15]—that the SMART program was able to help shift social norms for health-related behaviors. The SMART intervention also capitalized on group processes with a proven ability to generate behavior change [39]. CHWs organized women into meaningful cohorts for group sessions so that, for example, newly pregnant women or mothers of poorly nourished children could share successful strategies and offer mutual support for behavior change. In essence, the SMART intervention worked to enhance various forms of social capital, including close ties between family and friends (bonding capital), ties with neighbors and CHWs (bridging capital), and ties with village authorities and doctors (linking capital) [11]. Although the SMART intervention did not explicitly use a social capital framework, some studies suggest that this approach may be an effective way to shift behavioral norms, diffuse information, and promote health [12,40].

However, it is difficult to assess the extent to which this broader approach—reaching beyond individual women to family and community members—enhanced the effectiveness of the intervention. The program evaluation was designed only to measure outcomes at the individual level, not at the household or community levels. Although some focus groups were conducted with a convenience sample of family members, CHWs, and local health care providers after the project ended, their number and scope were limited [15]. Thus, an important opportunity was missed to measure the scope and impact of the project’s engagement with household members and the community.

### Feasibility and Sustainability of the SMART Approach

Experience with community-led women’s groups in Bangladesh [41] and a community-based perinatal and newborn preventive care package in Pakistan [37] has demonstrated that community-based health promotion activities can be a low-cost, feasible, and sustainable approach to changing health knowledge and practice in areas where the health system has limited resources. Coupling this kind of community-based approach together with partnerships between the government and non-state organizations has been a major contributor to improved health service coverage and health outcomes in Bangladesh [42]. The SMART project successfully employed these same strategies.

Reliance on existing civil society structures with strong ties in the community distinguishes the SMART approach and was a major key to its success. The experience in Egypt—in which 112 CDAs reached 2 million people in a just over one year—shows that it is feasible to bring effective interventions to large populations by working through local organizations with a strong community outreach component. The CDAs’ and CHWs’ roots in the community encouraged active community engagement with and broad support for SMART activities [43,44]. Working through CDAs also helped diversify investments in health, relieving the burden on the public health system, and proved especially valuable during a time of political instability and disruptions in public sector services. For example, some CDAs extended coverage of SMART activities to remote villages beyond the program’s target areas and mobilized additional resources to make essential medications and commodities such as iron folate available for free or at low cost to mothers and children [15]. The SMART program also benefitted from considerable worldwide experience with CHWs, which has demonstrated the importance of thoughtful design and implementation of recruitment, training, supervision, and support processes [43,45,46]. Hallmarks of the SMART approach included a transparent hiring process that resisted political pressures, substantial investments in training and supervision, and community and peer support systems to promote teamwork, morale, and retention.

Relying on CDAs with strong ties to and a permanent presence in the community—and investing in building their capacity—is contributing to the long-term sustainability of SMART activities. Umbrella CDAs took ownership of the intervention: they adjusted it to fit the local setting, identified local implementing partners, and found replacements when local CDAs did not perform as expected. When the program ended, CDAs had the managerial and financial capacity to continue supporting the intervention. Many used internal funding sources, such as membership dues and charitable donations, to continue paying CHWs and/or expand the intervention into new areas. Using grant-writing skills acquired during the program, 47 CDAs raised more than $7 million to fund health-related activities after the program ended [14]. This amount far exceeds the $2.5 million the project distributed to the 100 participating CDAs and comes close to the $10 million total direct and indirect costs of the project, which included training, capacity-building, financial and operational management, technical assistance, and materials. Because CDAs are present in every village in Egypt, scaling up the intervention is quite feasible.

However, the success of the SMART approach depends, in part, on the setting, as the weaker impact in Lower Egypt demonstrates. It requires a network of well-established, indigenous non-governmental organizations that are engaged with and have the support of the community, have the capacity to manage and support local implementing partners, and have a positive relationship with the government [47]. There must be a pool of motivated women who are able and willing to work long hours as CHWs for limited compensation. Finally, the approach works best where there is a strong sense of community and appreciation of the importance of giving back to the community, making it especially well-suited to rural areas and faith-based non-governmental organizations.

### Strengths and Limitations

Although we employed a rigorous evaluation design, it is possible that the findings underestimate the impact of the SMART intervention because of simultaneous programming at comparison sites. During the intervention period, Save the Children, Plan International, and UNICEF implemented activities to improve family health in Asyut that were similar to the SMART package. This may have had a disproportionate effect on the results, because Asyut accounted for the majority of survey respondents in the comparison group.

The study also has some limitations. The baseline questionnaire did not ask about the number of children or wealth-related items, so the multivariate analysis could not control for these characteristics. The significant difference in wealth between intervention and comparison groups at endline raises concerns, but the nature of the analysis reduces the potential for bias. The analysis does not compare endline levels of knowledge and behavior in the intervention and comparison groups. Instead it compares changes over time within each group. Data on some behaviors are based on women’s recall and may be subject to bias. The findings may not be generalizable across the entire country because researchers excluded some insecure areas and because urban areas were under-represented.

Finally, the duration of the intervention was too short to expect to see changes in health impacts, so this evaluation is limited to changes in knowledge and behavior. Data management challenges made it difficult to measure program outcomes, and some rare behaviors (e.g., seeking care from a local doctor after identifying a danger sign) could not be measured. One of the lessons learned from this experience is the need to build a reliable and easy to use monitoring and evaluation system that permits reliable data reporting, compilation, and analysis. In the Egyptian context, the best solution might be an electronic data collection system that lets CHWs submit data via tablets as they conduct activities.

## Conclusion

Despite increased demand for and use of maternal and child health services in Egypt, many women still do not have access to the information, guidance, and support they need to maintain the health of their families. We have demonstrated the ability of local civil society organizations with minimal health programming experience to bring effective counseling to pregnant women and new mothers via CHWs, increase knowledge, and promote behaviors associated with better health outcomes. The success of the SMART intervention is especially impressive given its short duration, CDAs’ and CHWs’ lack of experience in the health sector, the minimal inputs made to improve health services, and the civil unrest and disruptions in public services that occurred during the intervention period. Given the positive results in Egypt, donors should consider increasing support to civil society organizations that will work hand-in-hand with public and private service providers to fill gaps in health education and counseling and to marshal community support for behavior change.
